# The significance of phosphorylated heat shock protein 27 on the prognosis of pancreatic cancer

**DOI:** 10.18632/oncotarget.7424

**Published:** 2016-02-16

**Authors:** Mitsuru Okuno, Ichiro Yasuda, Seiji Adachi, Masanori Nakashima, Junji Kawaguchi, Shinpei Doi, Takuji Iwashita, Yoshinobu Hirose, Osamu Kozawa, Naoki Yoshimi, Masahito Shimizu, Hisataka Moriwaki

**Affiliations:** ^1^ Department of Gastroenterology, Gifu University Graduate School of Medicine, Gifu, 501-1194, Japan; ^2^ Department of Pathology, Osaka Medical College, Takatsuki, Osaka, 569-8686, Japan; ^3^ Department of Pharmacology, Gifu University Graduate School of Medicine, Gifu, 501-1194, Japan; ^4^ Department of Pathology and Oncology, Graduate School of Medical Science, University of the Ryukyus, Nishihara-cho, Okinawa, 903-0215, Japan

**Keywords:** HSP27, phosphorylation, pancreatic cancer, prognosis

## Abstract

**Background and Aim:**

The precise role of phosphorylated heat shock protein (HSP) 27 (p-HSP27) in pancreatic cancer remains to be elucidated. The aim of this study was to investigate whether the expression of p-HSP27 predicts the prognosis of patients with pancreatic cancer.

**Methods:**

We retrospectively assessed 49 biopsied pancreatic cancer tissue samples that were obtained prior to the treatment with gemcitabine. The correlations between p-HSP27 and the clinicopathological characteristics were analyzed.

**Results:**

p-HSP27 was not correlated with the response to chemotherapy or histological type. However, the median survival time was significantly longer in the patients with high p-HSP27 (275 days, *n* = 18) than those with low p-HSP27 (205 days, *n* = 31) (*P* = 0.0158). A multivariate Cox proportional hazards regression analysis revealed that low p-HSP27 predicted a worse prognosis.

**Conclusions:**

Higher p-HSP27 expression before chemotherapy was correlated with better survival, indicating that p-HSP27 expression could be used to predict the prognosis of pancreatic cancer.

## INTRODUCTION

Pancreatic cancer, which causes approximately 266,000 deaths per year, is the eighth leading cause of cancer-related deaths worldwide [1]. Surgical resection is the only curative treatment for pancreatic cancer; however only 10–15% of patients are eligible for surgery at the time of diagnosis. Chemotherapy is therefore important for the management of this malignancy. The clinical response and survival achieved by the administration of gemcitabine, a chemotherapeutic agent that is used in the treatment of pancreatic cancer, is superior to that achieved with 5-fluorouracil [2]. Gemcitabine, a nucleoside analog of deoxycytidine that inhibits DNA synthesis and induces apoptosis [3], is therefore a key anticancer drug for the treatment of pancreatic cancer. Gemcitabine-induced apoptosis involves the activation of p38 mitogen-activated protein kinase (MAPK) [4].

Heat shock proteins (HSPs) were first discovered as a family of proteins that are induced by heat shock, and other chemical and physical stresses [5]. HSPs act as molecular chaperones which prevent the aggregation of proteins and keep them in a folding state [6]. HSPs are generally recognized to play a crucial role in cell survival under stress conditions. HSPs are currently classified, according to their molecular mass, into seven major families including HSPA (HSP70), HSPB (small HSPs), HSPC (HSP90) and HSPH (HSP110) [7]. HSP27 (HSPB1), which is a member of the small HSPs (HSPs with monomer molecular masses in the range of 12–43 kDa), was identified as an inhibitor of actin polymerization and is ubiquitously expressed in various tissues [8]. HSP27 is able to bind to improperly folded proteins and further transfer them to ATP-dependent chaperones such as HSPA (HSP70) or to the protein degradation machinery, which includes the proteasomes or autophagosomes.

HSP27 undergoes various types of post-translational modifications such as phosphorylation, in which the cellular functions of HSP27 are modulated [9]. Human HSP27 is mainly phosphorylated at three sites (Ser-15, Ser-78, and Ser-82), and the phosphorylation is catalyzed by various protein kinases, including MAPK activated protein kinase 2 (MAPKAPK-2) [10], which is directly activated by p38 MAPK [11]. Unphosphorylated HSP27 forms large aggregated oligomers while its phosphorylation results in conformational changes that lead to small dissociated oligomers [9]. Phosphorylated HSP27 (p-HSP27) is reportedly implicated in tumor suppression and resistance to chemotherapy in various types of cancer [12–15]. In patients with pancreatic cancer, HSP27 expression is related to higher resistance to gemcitabine [16]. The gemcitabine-induced phosphorylation of HSP27 is also associated with this resistance [17].

On the other hand, we previously reported that p-HSP27, which is induced by gemcitabine via p38MAPK-MAPKAPK-2, plays a key role in the suppression of pancreatic cancer cell growth in stably transfected-mutant HSP27 pancreatic cancer cell lines [9]. In the present study, we investigated the relationship between a positive p-HSP27 ratio and subsequent treatment outcomes with gemcitabine, using pancreatic cancer tissues which were obtained by endoscopic ultrasound-guided fine-needle aspiration (EUS-FNA).

## RESULTS

### HSP27 expression level score and positive p-HSP27 ratio

**Figure 1:**
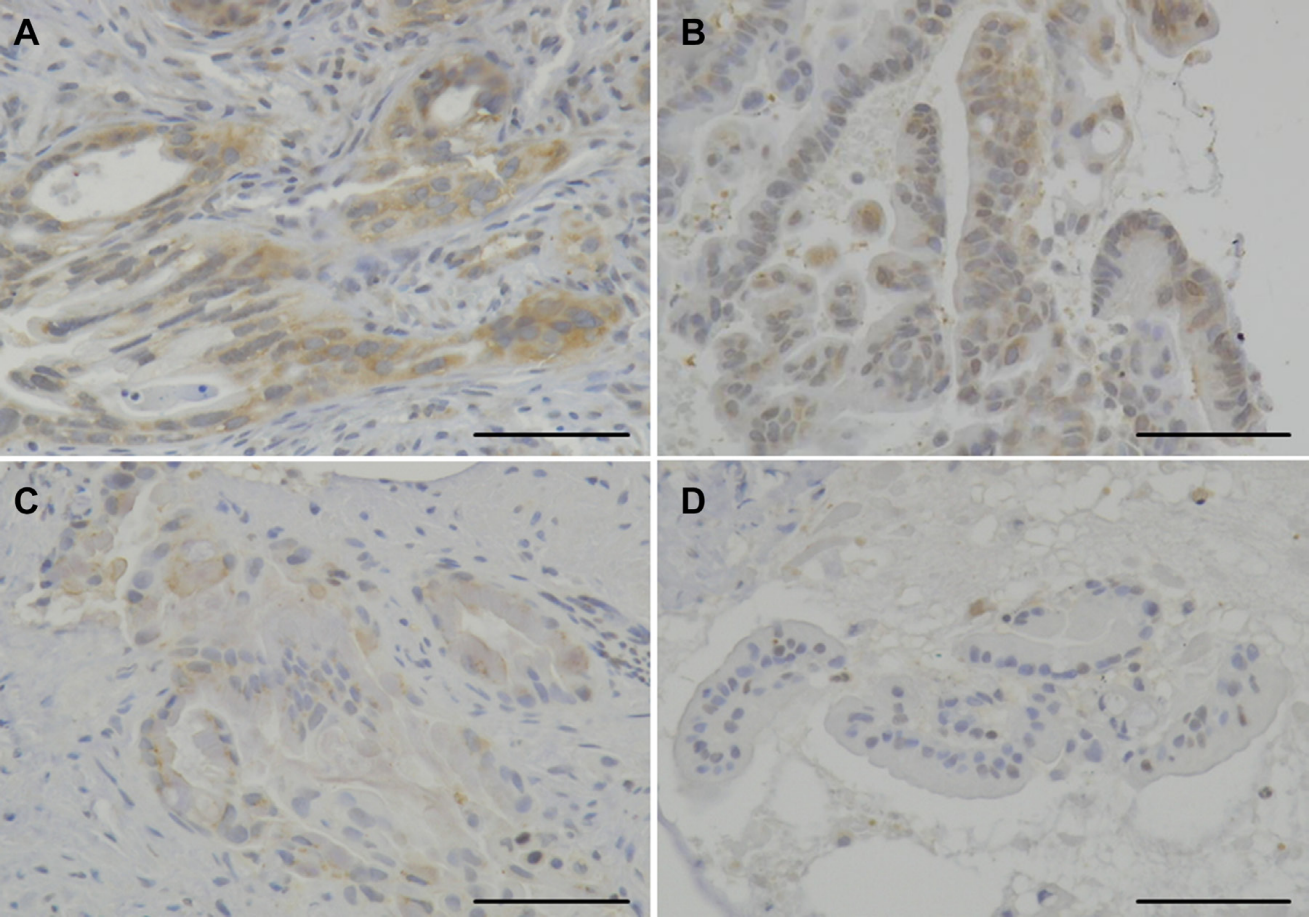
The immunohistochemical analysis for HSP27 The expression level score of HSP27 in patients with pancreatic cancer was highly case-dependent. Representative staining panels for the grade +3 (**A**), +2 (**B**), +1 (**C**), and 0 (**D**) cases are shown (original magnification: ×200). Scale bars indicate 100 μm.

**Figure 2:**
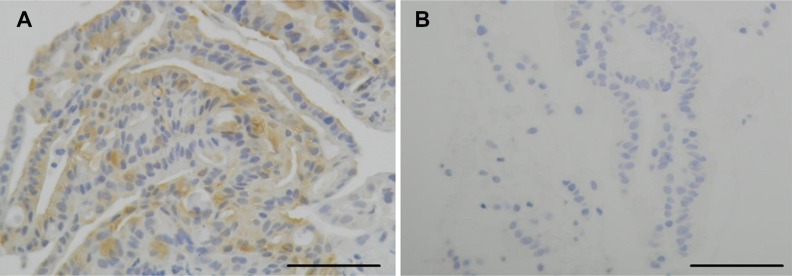
The immunohistochemical analysis for phosphorylated HSP27 (p-HSP27) The positive p-HSP27 ratio in patients with pancreatic cancer was also case-dependent. Representative staining panels for the p-HSP27-positive (**A**) and p-HSP27-negative (**B**) cases are shown (original magnification: ×200). Scale bars indicate 100 μm.

We retrospectively analyzed the HSP27 expression level score and positive p-HSP27 ratios in biopsy specimens obtained by EUS-FNA. While the positive expression of HSP27 was found in all of the 49 patients (100%), the intensity of the HSP27 staining differed considerably among the cases (Figure 1). We therefore employed numerical grading (0, +1, +2 and +3), as shown in Figure 1, and provided the HSP27 expression level score for each patient. In contrast, a positive p-HSP27 ratio was detected in the in pancreatic cancer cells of 47 (96%) cases (Figure 2).

**Figure 3:**
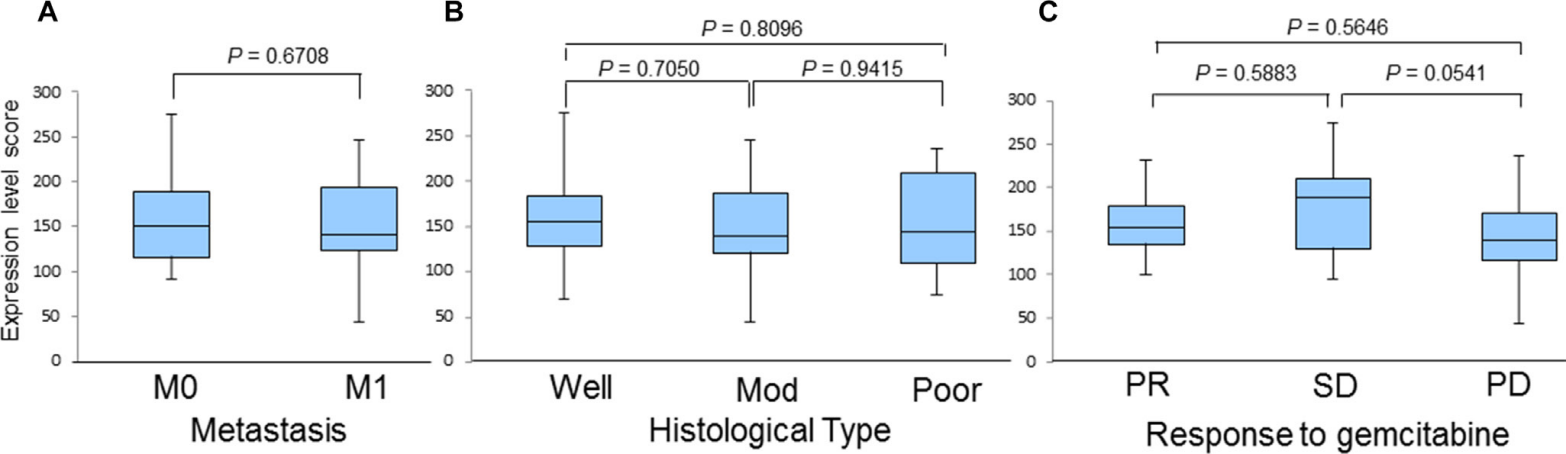
A comparison of HSP27 expression level scores in patients based on the presence or absence of metastasis (A), histological type (B), and response to gemcitabine-based chemotherapy (C) Abbreviations: M0, distant metastasis (−); M1, distant metastasis (+); Well, well-differentiated adenocarcinoma; Mod, moderately differentiated adenocarcinoma; Poor, poorly differentiated adenocarcinoma; PR, partial response; SD, stable disease; PD, progressive disease.

**Figure 4:**
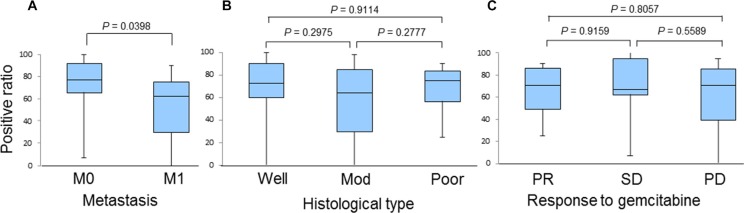
A comparison of the positive p-HSP27 ratios in patients based on the presence or absence of metastasis (A), histological type (B), and response to gemcitabine-based chemotherapy (C) Abbreviations: M0: distant metastasis (−); M1, distant metastasis (+); Well, well-differentiated adenocarcinoma; Mod, moderately differentiated adenocarcinoma; Poor, poorly differentiated adenocarcinoma; PR, partial response; SD, stable disease; PD, progressive disease.

We also investigated the correlation of the HSP27 expression level score with various factors, including the presence or absence of metastasis, histological type, and the response to chemotherapy (Figure 3). However, we did not find any significant differences, although the scores of patients with SD tended to be higher than those with PD (190 [95–275] and 140 [45–235], *P* = 0.0541; Figure 3C). We next examined the correlation between a positive p-HSP27 ratio and the patients' clinical characteristics. There were no significant differences based on histological type or the response to chemotherapy (Figure 4B and 4C). However, patients without metastasis (M0) were found to have a significantly higher positive p-HSP27 ratio than those with metastasis (M1; 77.5 [7.5–100] and 62.5 [0–90]; *P* = 0.0398; Figure 4A).

### Patient survival

**Figure 5:**
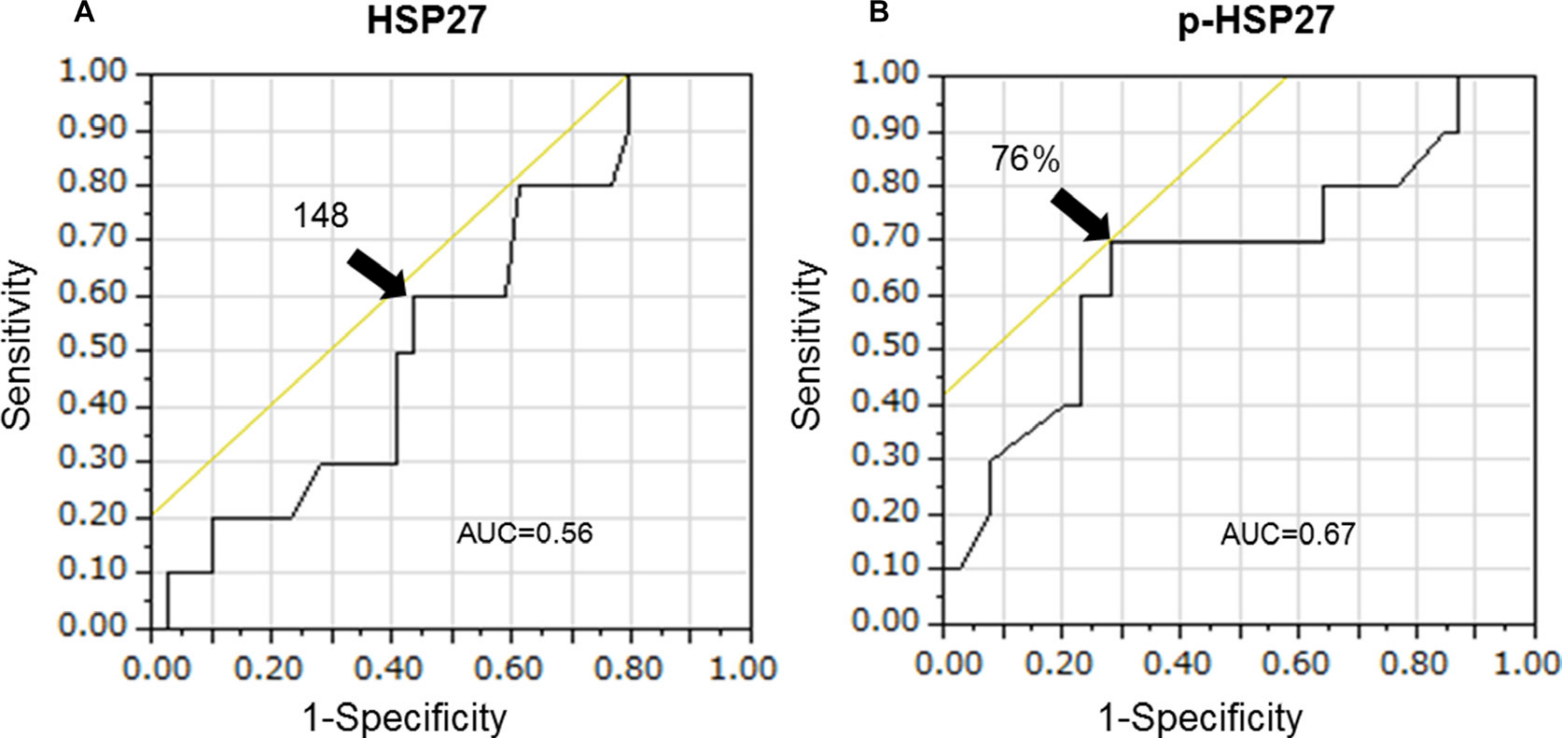
The area under the curve (AUC) values for the HSP27 expression level score (A) and a positive p-HSP27 ratio (B) are 0.56 and 0.67, respectively

We investigated whether the baseline characteristics of the enrolled patients and their HSP27 expression level score or positive p-HSP27 ratio were associated with survival in a univariate analysis using a log-rank test. The patients were divided into two groups according to the median age. When the patients were divided into two groups according to their survival period (more than 1 year or less than one year) in an ROC analysis, the area under the curve (AUC) values for the HSP27 expression level score and a positive p-HSP27 ratio were 0.56 and 0.67, respectively (Figure 5). The optimal cutoffs (%) were calculated by determining the smallest distance between the ROC curve and the upper-left corner of the graph (148 for the HSP27 expression level score and 76% for a positive p-HSP27 ratio). The sensitivity and specificity of these cut-off values were 60% and 56% for the HSP27 expression level score, and 70% and 72% for a positive p-HSP27 ratio, respectively. When the cutoff value was set at 148 for the HSP27 expression level score, there was no significant difference (low [< 148] and high [≤ 148], median survival periods: 223 [11–651] and 268 [106–790] days; *P* = 0.1186). However, when the cut-off value was set at 76% for a positive p-HSP27 ratio, there was a significant difference (low [< 76%] and high [≤ 76%], 205 [11–651] and 275 [110–790] days; *P* = 0.0158).

**Figure 6:**
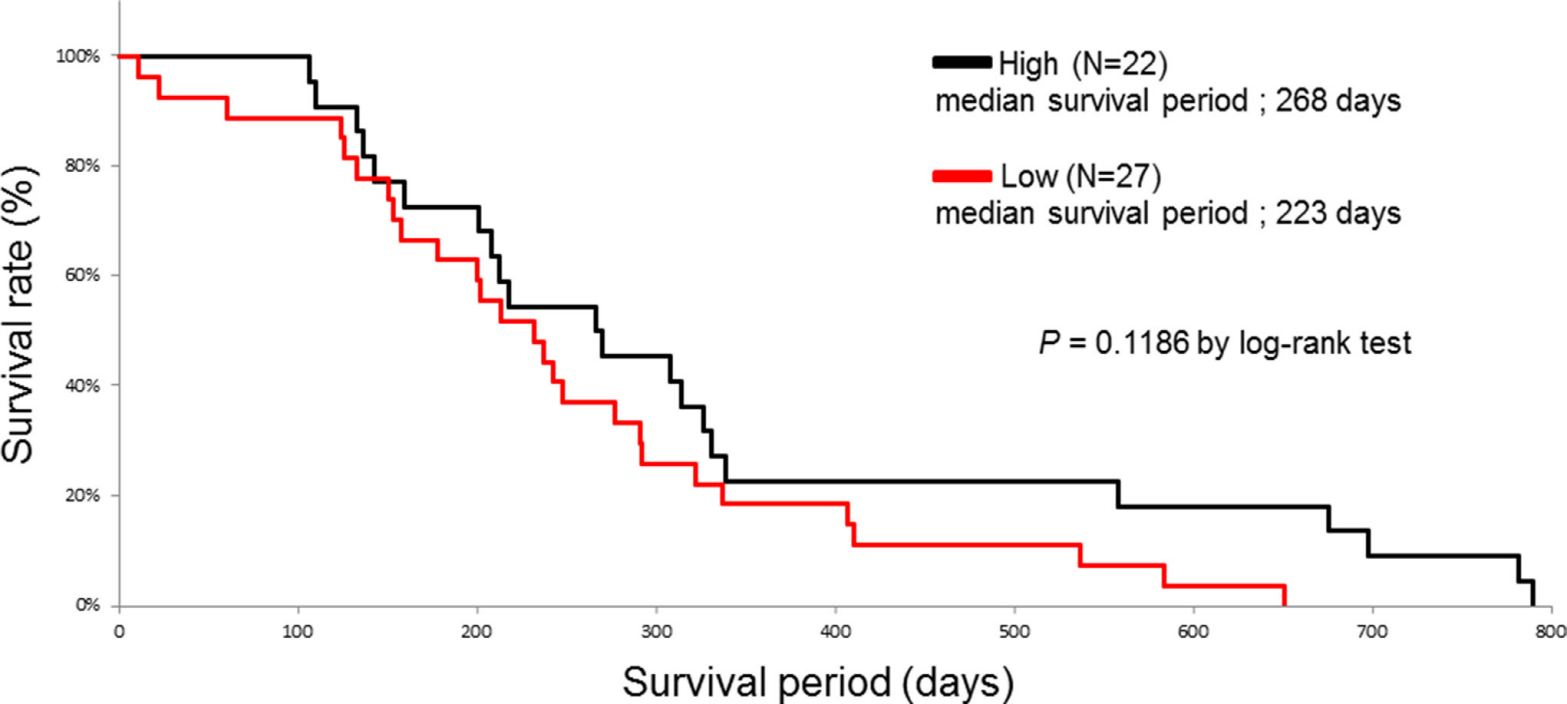
The Kaplan-Meier curves for the survival of pancreatic cancer patients with high (black line) and low (red line) HSP27 expression level scores

**Figure 7:**
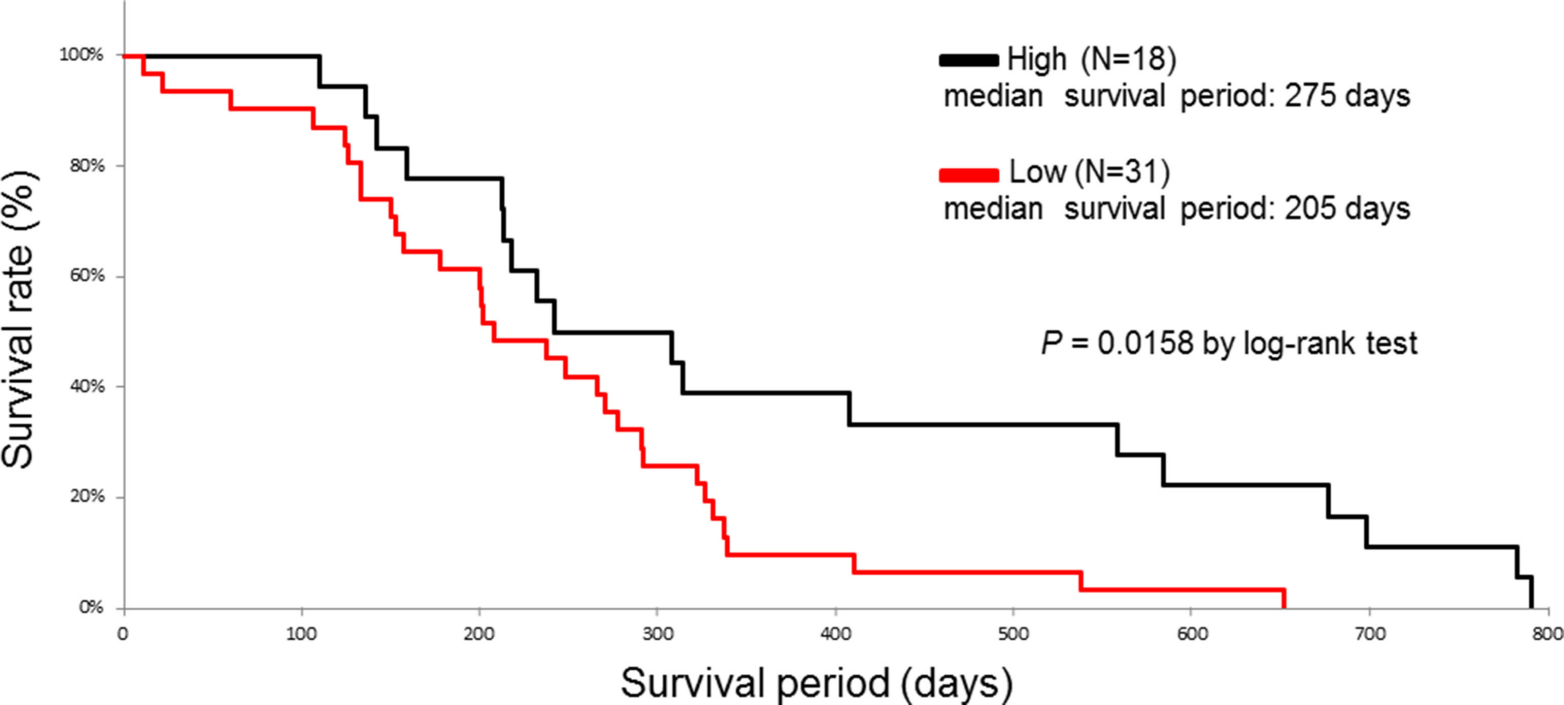
The Kaplan-Meier curves for the survival of pancreatic cancer patients with high (black line) and low (red line) positive p-HSP27 ratios

Sex (male and female, 205 [11–698] and 285 [124–790] days; *P* = 0.0408), metastasis (M0 and M1, 285 [11–790] and 205 [22–584] days; *P* = 0.0133), histological type (poor) (poor and well-mod, 180 [124–292] and 274 [11–790] days; *P* = 0.0016), and the response to chemotherapy (PD) (PD and PR-SD, 207 [11–584] and 303 [133–790] days; *P* = 0.0116) also remained to be significant predictors for overall survival (Table 1, Figures 6 and 7).

**Table 1:** The univariate analysis of the risk factors predicting the survival period (log-rank test)

Factors	Median survival (day)	*P*
**Sex**		
Male	205 (11–698)	
Female	285 (124–790)	0.0408
**Age**		
< 68	235 (11–651)	
≥ 68	230 (60–790)	0.2810
**HSP27 expression level score**		
Low	223 (11–651)	
High	268 (106–790)	0.1186
**Positive ratio of p-HSP27**		
Low	205 (11–651)	
High	275 (110–790)	0.0158
**Metastasis**		
M0	285 (11–790)	
M1	205 (22–584)	0.0133
**Histological type**		
Poor	180 (124–292)	
Well-Mod	274 (11–790)	0.0016
**Response to Chemotherapy**		
PD	207 (11–584)	
PR–SD	303 (133–790)	0.0116

Abbreviations: p-HSP27: Phosphorylated HSP27

M0: Distant metastasis (−)

M1: Distant metastasis (+)

Well: Well-differentiated adenocarcinoma

Mod: Moderately differentiated adenocarcinoma

Poor: Poorly differentiated adenocarcinoma

PR: Partial response

SD: Stable disease

PD: Progressive disease.

Moreover, we examined these variables using forward, stepwise selection, and found that a positive p-HSP27 ratio, sex, histological type (poor), and the response to chemotherapy (PD) were significant (data not shown). A multivariate Cox proportional hazards regression analysis was therefore performed for these factors. As a result, a low positive p-HSP27 ratio (hazard ratio [HR] = 2.135; 95% confidence interval (CI) = 1.089–4.523; *P* = 0.0265) and the histological type of poorly differentiated adenocarcinoma (HR = 2.972; 95% CI = 1.309–6.470; *P* = 0.0103) were found to be independently correlated with worse survival (Table 2).

**Table 2:** The multivariate analysis of the risk factors for predicting the survival period (cox proportional hazards regression model)

Factors	*P*	Hazard ratio	95% Confidence interval
Positive ratio of p-HSP27 (low)	0.0265	2.135	1.089–4.523
Histological type (poor)	0.0103	2.972	1.309–6.470

Abbreviations: p-HSP27: Phosphorylated HSP27

Poor: Poorly differentiated adenocarcinoma.

## DISCUSSION

The results of the present study showed the first evidence that a low positive p-HSP27 ratio, which was observed prior to treatment with gemcitabine-based chemotherapy, was significantly correlated with the presence of metastasis (Figure 4) and poor survival (Figure 7 and Table 2), while the HSP27 expression level score alone was not associated with the presence or absence of metastasis, histological type, response to chemotherapy or patient survival (Figures 3 and 6). A recent report showed that increased HSP27 expression might contribute to gemcitabine resistance [16]; however, another report indicated that there was no significant correlation between HSP27 expression and overall survival in patients with pancreatic cancer [18]. In our study, the patients who achieved SD tended to show a higher level of HSP27 expression than those with PD (Figure 3C). However, our findings are not completely consistent with previous studies, and thus, it is essential to accumulate an increased number of cases to elucidate the precise role of HSP27 in the response to chemotherapy.

A question arises here concerning the biological meaning and the mechanism underlying the superior survival of pancreatic cancer patients with increased levels of p-HSP27. Because p-HSP27 is associated with the induction of apoptosis in pancreatic cancer cells [9], the increased expression of p-HSP27 might be beneficial for suppressing the growth of these cells. We presume that increased p-HSP27 levels are involved in the activation of p38 MAPK, which induces apoptosis in pancreatic cancer cells [9], because functional p38 MAPK activity significantly contributes to improved survival in patients with pancreatic cancer [19]. The importance of HSP27 phosphorylation and p38 MAPK activation in the suppression of hepatoma cell growth has also been reported [13].

In the present study, the status of HSP27 phosphorylation before chemotherapy was not associated with the response to gemcitabine, although a previous study suggested that p-HSP27 might contribute to gemcitabine resistance [17]. In addition, the prognosis of the patients who express p-HSP27 was significantly improved (Figure 7). One of the reasons for this result is that presence of metastasis was lower in p-HSP27 positive patients (Figure 4A). The role of HSP27 phosphorylation in the suppression of invasion and metastasis in pancreatic cancer remains unclear and further studies should be performed to elucidate this point.

In addition, it might also be important to determine whether the expression levels of HSP27 and p-HSP27 were altered after gemcitabine-based chemotherapy. There is a possibility that gemcitabine sensitivity and the induction of apoptosis are increased at the pancreatic tissue level when a high positive p-HSP27 ratio is present [9]. It is most likely that the apoptosis of pancreatic cancer is more frequently induced in the patients with a high p-HSP27 ratio after gemcitabine therapy than in those with a low p-HSP27 ratio, and that this might be the reason for the difference in their respective survival periods. Evaluating the ratio of p-HSP27 to nonphosphorylated HSP before and after chemotherapy is also important because this ratio might be able to determine the boundary between survival and death in gemcitabine-resistance pancreatic cancer cells [20].

In conclusion, a positive p-HSP27 ratio was significantly associated with a longer survival time. In the near future, p-HSP27 could therefore be used as a prognostic marker for patients with pancreatic cancer.

## MATERIALS AND METHODS

### Patients' characteristics

The study population included 49 pancreatic cancer patients (female, *n* = 22; male, *n* = 27; median age, 68 years [range: 31–86 years]) who underwent EUS-FNA at the department of Gastroenterology, Gifu University Hospital between September 2004 and October 2011. The patients' demographic and clinicopathological characteristics are shown in Table 3. Metastasis was recognized in 27 patients (55%). Nineteen (39%) cases were well-differentiated (well), 19 (39%) were moderately differentiated (mod), and 11 (22%) cases were poorly differentiated adenocarcinoma (poor). All of the patients received gemcitabine-based chemotherapy. Their treatment responses included a partial response (PR) in 4 cases (8%), stable disease (SD) in 13 cases (27%), and progressive disease (PD) in 32 cases (65%).

**Table 3:** The baseline characteristics of the enrolled patients

Total number	49
Female/male	22 / 27
Age (years), median (range)	68 (31–86)
**Metastasis**	
M0	22
M1	27
**Pathological diagnosis**	
Well-differentiated adenocarcinoma	19
Moderately differentiated adenocarcinoma	19
Poorly differentiated adenocarcinoma	11
**Treatment**	
Surgical resection	0
Chemotherapy (GEM-based)	49
**Response to chemotherapy**	
PR	4
SD	13
PD	32

Abbreviations: M0: Distant metastasis (−)

M1: Distant metastasis (+)

PR: Partial response

SD: Stable disease

PD: Progressive disease.

The study protocol was approved by the review board for human research at Gifu University Hospital and was conducted in accordance with the human and ethical principles of research set forth by the Declaration of Helsinki.

### EUS-FNA

EUS was performed using an oblique forward-viewing electronic linear scanning video echoendoscope equipped with an elevator and a working channel of 2.8 mm in diameter (GF-UC240P-AL5; Olympus, Co, Ltd, Tokyo, Japan). The echoendoscope was connected to a processor with a color Doppler function (SSD-5000; Aloka Co, Ltd, Tokyo, Japan). FNA was performed with a 19-, 22- or 25-gauge needle (EchoTip; Wilson-Cook, Winston-Salem, NC, USA) under direct EUS guidance.

### Immunohistochemical analysis

The immunohistochemical staining of the biopsied tissues was performed with the streptavidin-biotin complex method to investigate the levels of HSP27 and p-HSP27, and their localization. The primary antibodies were anti-HSP27 and anti-p-HSP27 (Ser-82) antibodies, respectively (Santa Cruz Biotechnology, Santa Cruz, CA, USA). Briefly, deparaffinized sections were first treated with 3% H_2_O_2_ in methanol for 10 min to inhibit endogenous peroxidase activity. Sections were then immersed in 50 mM citrate buffer (pH 6.0), and heated in a microwave oven for 1 min. Next, each section was sequentially treated with biotinylated secondary antibodies and streptavidin-peroxidase complex (Dako Chem Mate, Kyoto, Japan). Finally, the immune complexes were visualized with 3, 3′-diaminobenzidine tetrahydrochloride as a chromogen. Mayer's hematoxylin was used as a counterstaining agent.

### Pathological evaluation

HSP27 expression level score: We calculated the HSP27 expression level score using the quantitative-histogram scoring method [21]. We graded the density of immunostaining from 0 (without staining, to +3 (the densest staining) (Figure 1). We then multiplied the grade by the % area on each specimen, and summed these products to give an expression level score ranging from 0 to 300. Positive p-HSP27 ratio: We measured the area with a positive signal on each biopsied specimen, and calculated its ratio in the cancerous area (Figure 2).

### Statistical analysis

All analyses were conducted using the JMP^®^ 8.0 software program (SAS Institute Inc., Cary, NC, USA). The values are expressed as the number of patients or the median (range). The median values were compared using Student's *t*-test. A receiver operating characteristic (ROC) analysis was performed to determine the cutoff values for the HSP27 expression level score, the positive p-HSP27 ratio. Survival rates were calculated using the Kaplan-Meier method and the difference between the curves was analyzed using the log-rank test. A multivariate Cox proportional hazards regression analysis was performed to test prognostic significance. Forward stepwise selection was used to obtain an efficient predictive model. All *P* values were reported. *P* values of < 0.05 were considered to be statistically significant.
